# Changes in the fermentation products, taxonomic and functional profiles of microbiota during high-moisture sweet sorghum silage fermentation

**DOI:** 10.3389/fmicb.2022.967624

**Published:** 2022-08-01

**Authors:** Jie Zhao, Xue-Jing Yin, Si-Ran Wang, Jun-Feng Li, Zhi-Hao Dong, Tao Shao

**Affiliations:** Institute of Ensiling and Processing of grass, College of Agro-grassland Science, Nanjing Agricultural University, Nanjing, China

**Keywords:** sweet sorghum, fermentation quality, microbial community, functional profiles, silage

## Abstract

The purpose of this study was to evaluate the fermentation quality, microbial community, and functional shifts of sweet sorghum during ensiling. The high-moisture sweet sorghum (SS) was naturally ensiled for 1, 3, 7, 15, 30, and 60 days. After 60 days of ensiling, sweet sorghum silage (SSS) showed homolactic fermentation with absent butyric acid, low pH value, acceptable concentrations of propionic acid, ethanol, and ammonia nitrogen and high lactic acid concentration. *Acinetobacter*, *Sphingomonas,* and *Pseudomonas* were the advantage genera in SS. While, *Lactococcus*, *Weissella,* and *Pediococcus* were dominant in 3-day SSS and subsequently replaced by *Lactobacillus* in 60-day SSS. Spearman’s correlation heatmap showed that *Pediococcus* and *Leuconostoc* were negatively related to the pH value of SSS. There were great differences in the Kyoto Encyclopedia of Genes and Genomes (KEGG) functional profiles of SS and SSS. Ensiling process downregulated the metabolism of amino acid, energy, cofactors, and vitamins, but upregulated the metabolism of nucleotides and carbohydrates. Overall, next-generation sequencing in conjunction with KEGG functional prediction revealed the distinct differences in the initial and late phases of ensiling in terms of both community succession and functional shifts. The knowledge regarding bacterial community dynamics and functional shifts of SS during ensiling is important for understanding the fermentation mechanism and may contribute to the production of high-quality sweet sorghum silage.

## Introduction

In recent years, the demand for forages is increasing with the rapid development of animal husbandry. There is a need to find alternative forage to alleviate the supply pressure of silage maize. Sweet sorghum (*Sorghum bicolor* (L.) Moench) is a perennial economic or feed crop belonging to the genus *Sorghum* of Gramineae and native to eastern Africa. It has been extensively spread in more than 100 countries due to its characteristics in terms of high light, nitrogen and water use efficiency, and large biomass yield (Velmurugan et al., 2020). As a promising forage, sweet sorghum produces grass and sugar content twice as much as maize but requires less than a third of the water (Li et al., 2013). Bassam and Jakob (1997) reported that sweet sorghum can be harvested three to four times a year and each harvest can produce 160 tons of aboveground biomass per hectare. Sweet sorghum has the characteristics of most C4 plants, such as waterlogging, drought, high temperature, salt, and barren tolerance, so it can thrive in humid, semi-humid, and semi-arid areas (Davila-Gomez et al., 2011; Bhat, 2019). It is not only called “the Camel among crops” (Umakanth et al., 2019) but is also known as “a promising alternative forage to maize” (Colombini et al., 2015; Tao et al., 2020). To date, sweet sorghum is widely used for the production of sugar, biofuel, and fiber products, especially livestock feed.

It is generally recommended to harvest sorghum for silage from the late milk stage to the early dough stage (Black et al., 1980). Feedback from farms, however, indicated that the harvest time of sweet sorghum is often earlier or later than the above stage. In fact, not only sweet sorghum but also maize and other forages in practice have unstable harvest times (Comino et al., 2014). Harvesting outside the recommended maturity range is owing to adverse weather, insufficient harvest capacity, unavailability of harvesting equipment at the optimal harvest time, errors in selecting maturity stages of hybrids, or lack of moisture monitoring during the ripe stage (Comino et al., 2014; Windle et al., 2014). Unlike the high dry matter (DM) resulting from late harvests that can be solved by direct water addition, wilting low DM forage caused by early harvests additional increases the cost of labor or drying equipment. Given that sweet sorghum with low DM is directly ensiled in many instances, it is necessary to clarify the fermentative mechanism under high-moisture conditions, which is of great significance for the production of high-quality sweet sorghum silage.

Ensilage is a lactic acid bacteria (LAB)-driven anaerobic fermentation process involving complex microbial interactions and activities. On this basis, the microbial ecology involved in the silage process needs to be studied at great length (Keshri et al., 2019). To date, culture-based methods have been difficult to meet the need to fully understand the succession of microbial communities from forage to silage. Culture-independent technologies such as next-generation sequencing (NGS) enable researchers to explore changes in the microbial community involved in the ensiling process (McAllister et al., 2018). The NGS has identified comprehensive and crucial microbial information for an increasing number of silage studies, but the functional annotation of microbial communities is still largely unknown. Asshauer et al. (2015) stated that the characterization of phylogenetic and functional diversity was a key factor in microbial community analysis. Thus, the prediction of higher-order functional profiles underlying the silage microbial community will be highly beneficial and can be used as a supplement to NGS analysis (Kanehisa and Goto, 2000).

Based on this, this study aimed to evaluate the fermentation quality, microbial community, and functional shifts of high-moisture sweet sorghum (SS) during ensiling.

## Materials and methods

### Silage making

Sweet sorghum was planted in the Jianghuai Watershed Experimental Station (latitude 32.57°N, longitude 117.50°E, elevation 70.3 m, Anhui, China). The experimental field (30 m^2^) was equally separated into three blocks (replicates) and eight plots in each block were randomly chosen for silage making. After 12 weeks of growth, SS was harvested at the heading stage and chopped into 2 cm theoretical lengths by a feed cutter. After mixing thoroughly, the chopped SS was split into two parts for silage preparation and fresh material analysis, respectively. A total of 18 bags (6 ensiling time × 3 replicates per treatment) were prepared. Specifically, approximately 450 g of mixed material was packed into a pre-sterilized laboratory silo (polyethylene plastic bag with the size of 50 × 30 cm, CNON Packaging Products Co., Ltd., Zhejiang, China), sealed by an Aomitai DZD-400 vacuum sealer (Aomitai Technology Co., Ltd., Jiangsu, China), and ensiled under ambient temperature (27.5 ± 2.5°C) for 1, 3, 7, 15, 30, and 60 days.

### Chemical and microbial composition analyses

Before analyses, fresh SS or SS silage (SSS) was mixed thoroughly in an ethanol-sterilized container. About 300 g of SS or SSS was oven-dried at 105°C for 15 min (deactivation of enzymes) and 65°C for 48 h to determine DM content. Subsequently, the dried sample was ground by a laboratory knife mill equipped with a 1-mm screen. The water-soluble carbohydrate (WSC) content of SS or SSS was determined by the anthrone-sulfuric acid method (Thomas, 1977). The neutral detergent fiber (NDF) content and acid detergent fiber (ADF) content of SS were determined by an Ankom 200 fiber analyzer (Ankom Technology, NY, United States). The total nitrogen (TN) content of SS and SSS was determined by a Kjeltec 8200 Kjeldahl nitrogen analyzer (Foss Analytics, Höganäs, Sweden). The crude protein (CP) content of SS was calculated by multiplying the TN content by 6.25. The buffering capacity (BC) of SS was determined by titration (Playne and McDonald, 1966).

After extracting 20 g SS or SSS with 60 ml deionized water at 4°C for 24 h, the extract was filtered with four layers of sterile gauze and Whatman filter paper. Then, the pH of SS or SSS was determined by a Hanna HI 2221 pH meter (Hanna Instruments, Inc., Woonsocket, RI, United States). The ammonia nitrogen (NH_3_-N) concentration of SSS was determined by the phenol-hypochlorite method (Broderick and Kang, 1980). The concentrations of lactic acid (LA), acetic acid (AA), propionic acid (PA), n-butyric acid (n-BA), isobutyric acid (IBA), ethanol, and 1,2-propanediol of SSS were quantified by an Agilent 1,260 Infinity HPLC system (Agilent Technologies, Inc., Waldbronn, Germany) equipped with a refractive index detector (column: Carbomix^®^ H-NP5, Sepax Technologies, Inc., Newark, DE, United States; eluent: 2.5 mM H_2_SO_4_, 0.5 ml/min; temperature: 55°C), as reported in our previous study (Zhao et al., 2018). The concentration of volatile fatty acids (VFA) was the sum of LA, AA, PA, BA, and IBA concentrations.

About 10 g of SS or SSS was homogenized with 90 ml autoclaved 0.85% sodium chloride solution and shaken at 120 rpm, 37°C for 2 min. Subsequently, 1 ml above solution was serially diluted at a 10-fold gradient for microbial counting. The LAB, aerobic bacteria, yeasts, molds, and enterobacteria were counted by culture-based methods using de Man, Rogosa, and Sharp agar, nutrient agar, potato dextrose agar, and violet red bile glucose agar media, respectively (Zhao et al., 2021). The microbial number was enumerated in colony-forming units (CFU), converted to logarithmic form, and expressed on a fresh material (FM) basis. After filtration with two layers of sterile gauze, the obtained filtrate was collected for subsequent bacterial DNA extraction and stored at −80°C.

### The NGS analysis

The bacterial community changes greatly in the initial phase of ensiling, and the late phase of ensiling is critical for the final quality of silage. Thus, SS, 7-day SSS (SSS-7), and 60-day SSS (SSS-60) were sampled for the analyses of bacterial diversity and potential function. The bacterial DNA extraction and polymerase chain reaction (PCR) amplification were conducted according to the procedures reported in our previous study (Zhao et al., 2022). The purified amplicons were paired-end sequenced on the Illumina MiSeq PE300 platform (Illumina Inc., San Diego, CA, United States). The FLASH software (ver. 1.2.11) was selected to process all raw reads, and the QIIME software (ver. 1.9.1) was used to filter out the low-quality sequences with quality scores less than 20. The UCHIME algorithm was applied to remove chimeric sequences, and the UPARSE pipeline (ver. 7.0) was chosen to cluster the qualified reads with 97% identities into operational taxonomic units (OTUs). Based on the SILVA database (ver. 132), the above OTUs were classified and denominated at the phylum and genus levels using the RDP classifier (ver. 2.11) with a confidence threshold of 70%. The QIIME software was also used to calculate bacterial α-diversities including Shannon, Chao, Ace, Sobs, Simpson, and Coverage indexes as well as β-diversities presented by the Bray-Curtis metric. The Vegan package of R software (ver. 4.0.5) was loaded to construct principal co-ordinate analysis (PCoA) plot for visualizing the Bray-Curtis distance metric. The Pheatmap package was loaded to construct the Spearman’s correlation heatmap for visualizing the linkages between fermentation parameters and bacterial community. The sequenced data were deposited in the NCBI Sequence Read Archive database under BioProject PRJNA843380.

### Kyoto encyclopedia of genes and genomes (KEGG) functional prediction analysis

The functional profiles of the bacterial community in SS and SSS were predicted using the Tax4fun tool. First, the 16S rRNA profile based on the SILVA database was transformed into a taxonomic profile of prokaryotes based on the KEGG database. Then, the 16S rRNA copy number was applied to normalize the abundance of KEGG prokaryotes. Finally, the functional profiles from sequencing data were predicted by the linear combination of KEGG prokaryotes functional profiles and normalized taxonomic abundances (Asshauer et al., 2015).

### Statistical analysis

One-way ANOVA was applied to investigate the effects of ensiling time on fermentation parameters, chemical composition, microbial number, and α-diversity indexes of silages based on the general linear model of SAS (ver. 9.2; SAS Institute Inc., NC, United States). Meanwhile, the analysis of similarity (ANOSIM) with 10,000 permutations was adopted to statistically compare the difference in β-diversity of bacterial community, and the Wilcoxon rank-sum test was applied to analyze the differences in KEGG functional profiles. The statistical differences were significant at *p* < 0.05.

## Results

### Characteristics of fresh SS

Table 1 shows that the fresh SS had a DM content of 19.2% FM, WSC of 15.6% DM, and CP of 7.68% DM. The BC was 73.4 mEq/kg DM. The number of LAB, aerobic bacteria, yeasts, molds, and enterobacteria was 6.07, 7.64, 6.32, 5.09, and 6.46 log_10_ CFU/g FM, respectively.

**Table 1:** The chemical and microbial composition of fresh SS (means ± standard deviations).

Items	SS
pH	5.58 ± 0.06
DM (% FM)	19.2 ± 1.54
WSC (g/kg DM)	15.6 ± 0.56
CP (% DM)	7.68 ± 0.25
BC (mEq/kg DM)	73.4 ± 3.61
NDF (% DM)	49.2 ± 0.38
ADF (% DM)	25.5 ± 0.19
LAB (Log_10_ CFU/g FM)	6.07 ± 0.19
Aerobic bacteria (Log_10_ CFU/g FM)	7.64 ± 0.06
Yeasts (Log_10_ CFU/g FM)	6.32 ± 0.14
Molds (Log_10_ CFU/g FM)	5.09 ± 0.16
Enterobacteria (Log_10_ CFU/g FM)	6.46 ± 0.35

SS, high-moisture sweet sorghum; DM, dry matter; FM, fresh material; WSC, water-soluble carbohydrates; BC, buffering capacity; NDF, neutral detergent fiber; ADF, acid detergent fiber; CP, crude protein; LAB, lactic acid bacteria; CFU, colony-forming units.

### Fermentation quality of SSS

As shown in Table 2, ensiling time significantly (*p* < 0.05) affected the pH value, the concentrations of LA, AA, VFA, and NH_3_-N as well as the content of DM and WSC. The pH significantly (*p* < 0.05) decreased during the first 7 days of ensiling reaching the minimum of 3.96 on day 15 of ensiling, and then increased gradually (*p* > 0.05). The LA concentration exhibited the opposite trend to the pH value, with the highest concentration of 5.36% DM on day 30 of ensiling. The concentrations of AA, VFA, and NH_3_-N increased as ensiling proceed (*p* < 0.01). Low concentration of PA was detected throughout the ensiling process. No n-BA, IBA, or 1,2-propanediol was found in all SSS. As ensiling proceeded, the DM and WSC content significantly (*p* < 0.01) decreased.

**Table 2:** Changes in the chemical composition and fermentation quality of SSS.

Items	Ensiling time (d)						SEM	*p-*Values
	1	3	7	15	30	60		
pH	4.99^A^	4.48^AB^	3.94^B^	3.75^B^	3.91^B^	4.02^AB^	0.123	0.013
LA (% DM)	1.02^D^	1.72^CD^	2.30^CD^	3.27^BC^	5.36^A^	5.07^AB^	0.408	< 0.001
AA (% DM)	0.54^C^	0.66^C^	1.20^BC^	1.63^AB^	2.03^A^	2.26^A^	0.163	< 0.001
LA/AA	2.05	2.90^A^	2.16	2.03	2.67	2.25	0.149	0.518
PA (% DM)	0.11	0.07	0.04	0.06	0.15	0.19	0.021	0.295
n-BA (% DM)	ND	ND	ND	ND	ND	ND	–	–
IBA	ND	ND	ND	ND	ND	ND	–	–
VFA (% DM)	0.64^C^	0.74^C^	1.24^BC^	1.68^AB^	2.18^A^	2.45^A^	0.173	< 0.001
Ethanol (% DM)	0.02	0.02	ND	0.10	0.28	0.37	–	–
1,2-propanediol	ND	ND	ND	ND	ND	ND	–	–
DM (% FM)	18.7^A^	16.8^AB^	15.3^B^	14.2^B^	14.3^B^	14.9^B^	0.434	0.004
WSC (% DM)	13.2^A^	12.3^A^	11.3^AB^	9.26^BC^	6.94^C^	7.05^C^	0.607	< 0.001
NH_3_-N (% TN)	5.19^C^	6.54^ABC^	6.15^BC^	6.30^ABC^	6.93^AB^	7.59^A^	0.198	0.002

Means with different superscript letters differ at *p* < 0.05.

SS, SS silage; DM, dry matter; FM, fresh material; LA, lactic acid; AA, acetic acid; LA/AA, the ratio of lactic to acetic acid; PA, propionic acid; n-BA, n-butyric acid; WSC, water-soluble carbohydrates; NH_3_-N, ammonia nitrogen; TN, total nitrogen. SEM, standard error of means.

Ensiling time had significant (*p* < 0.001) effects on the number of LAB, aerobic bacteria, and yeasts (Table 3). The LAB number significantly (*p* < 0.001) increased to the maximum of 9.84 log_10_ CFU/g FM in the first 30 days of ensiling, then decreased significantly (*p* < 0.001). While the number of aerobic bacteria, yeast, molds, and enterobacteria constantly decreased to low or undetectable levels.

**Table 3:** Changes in the microbial number of SSS.

Items	Ensiling time (d)						SEM	*p-*Values
	1	3	7	15	30	60		
LAB (log_10_ CFU/g FM)	6.05^D^	8.87^BC^	9.29^ABC^	9.63^AB^	9.84^A^	8.48^C^	0.305	< 0.001
Aerobic bacteria (log_10_ CFU/g FM)	6.33^A^	5.69^AB^	3.40^BC^	3.66^BC^	< 2.00^C^	< 2.00^C^	0.448	< 0.001
Yeasts (log_10_ CFU/g FM)	5.95^A^	5.49^AB^	3.83^BC^	3.58^C^	3.50^C^	< 2.00^D^	0.311	< 0.001
Molds (log_10_ CFU/g FM)	4.20	3.63	< 2.00	ND	ND	ND	–	–
Enterobacteria (log_10_ CFU/g FM)	5.55	4.35	2.04	< 2.00	ND	ND	–	–

Means with different superscript letters differ at *p* < 0.05.

SS, SS silage; LAB, lactic acid bacteria; CFU, colony-forming units; FM, fresh material. SEM, standard error of means.

### The bacterial community of SS and SSS

**Figure 1:**
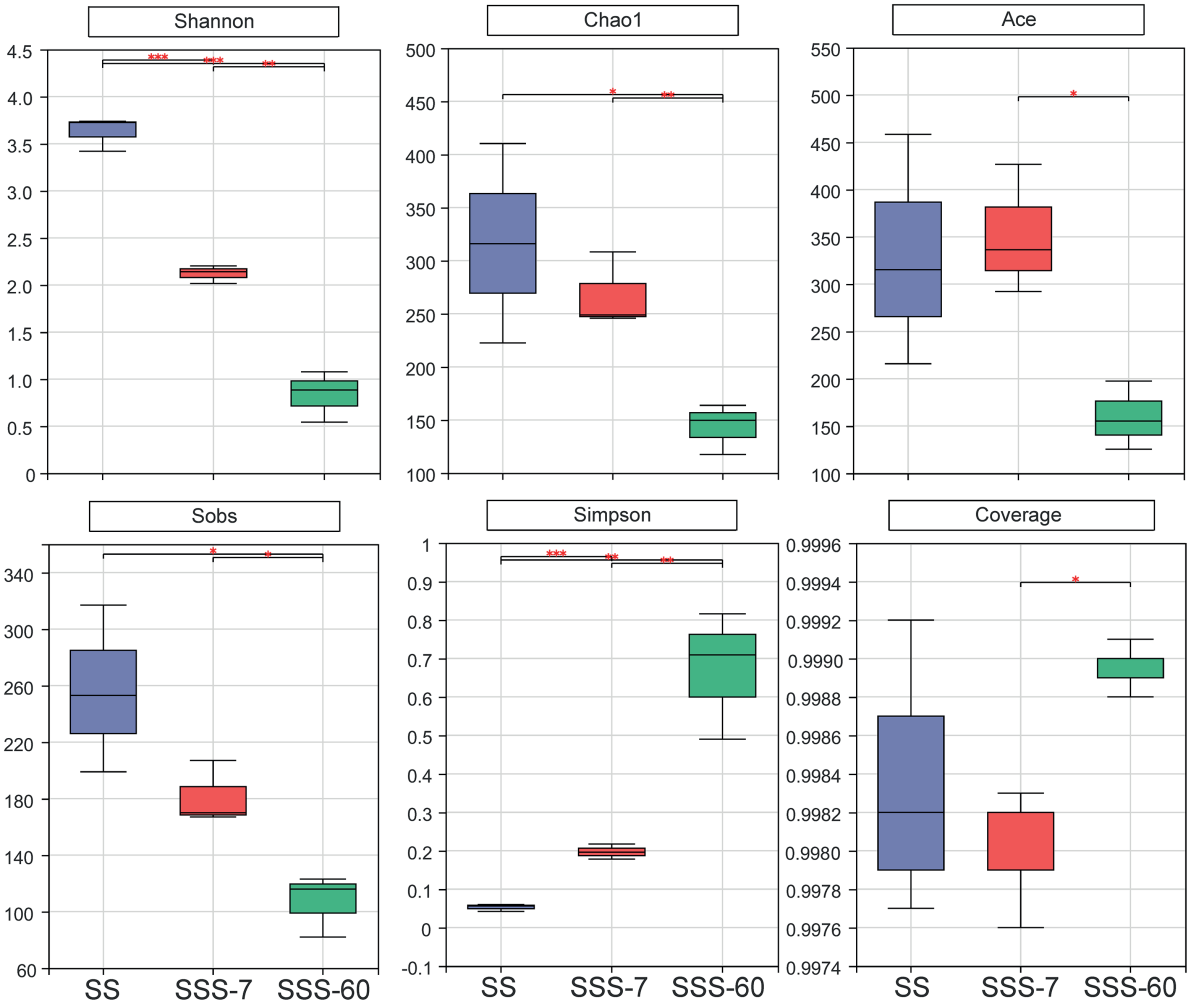
Bacterial *α*-diversities (Shannon, Chao1, Ace, Sobs, Simpsn, Coverage) of SS and SSS. SS, high-moisture sweet sorghum; SSS, SS silage. ^*^p < 0.05; ^**^0.001 < *p* ≤ 0.01; ^***^p ≤ 0.001.

**Figure 2:**
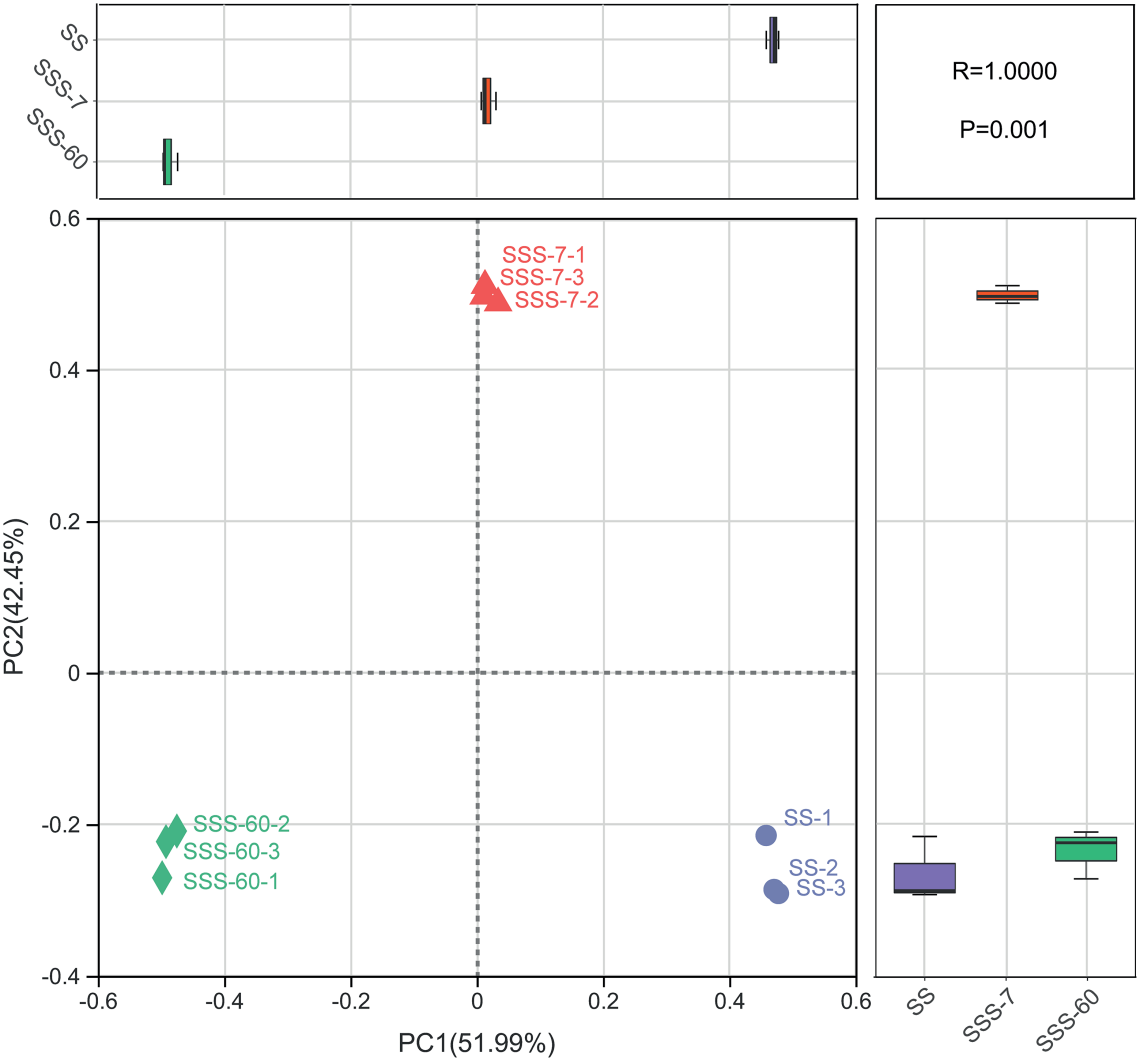
Bacterial β-diversity of fresh and ensiled EG, calculated by principal co-ordinates analysis (PCoA) plot based on the Bray–Curtis distance metric. SS, high-moisture sweet sorghum; SSS, SS silage.

The α-diversities of the bacterial community in SS and SSS are presented in Figure 1. α-diversity can be analyzed to reflect the richness (Chao1 and Sobs) and diversity (Shannon and Simpson) of microbial communities. The indexes of Shannon, Chao1, and Sobs were highest in SS, followed by SSS-7 and SSS-60, while the index of Simpson exhibited the opposite trend. The coverage index of all sequenced samples was above 0.995. The difference of β-diversities in the bacterial community of fresh and ensiled EG based on the Bray-Curtis distance metric is shown in Figure 2. β-diversitiy was analyzed to study the similarity or difference of microbial community structure in different treatments. A clear separation was observed among the symbols of SS, SSS-7, and SSS-60 in the PCoA plot. The symbols of each treatment were clustered together.

**Figure 3:**
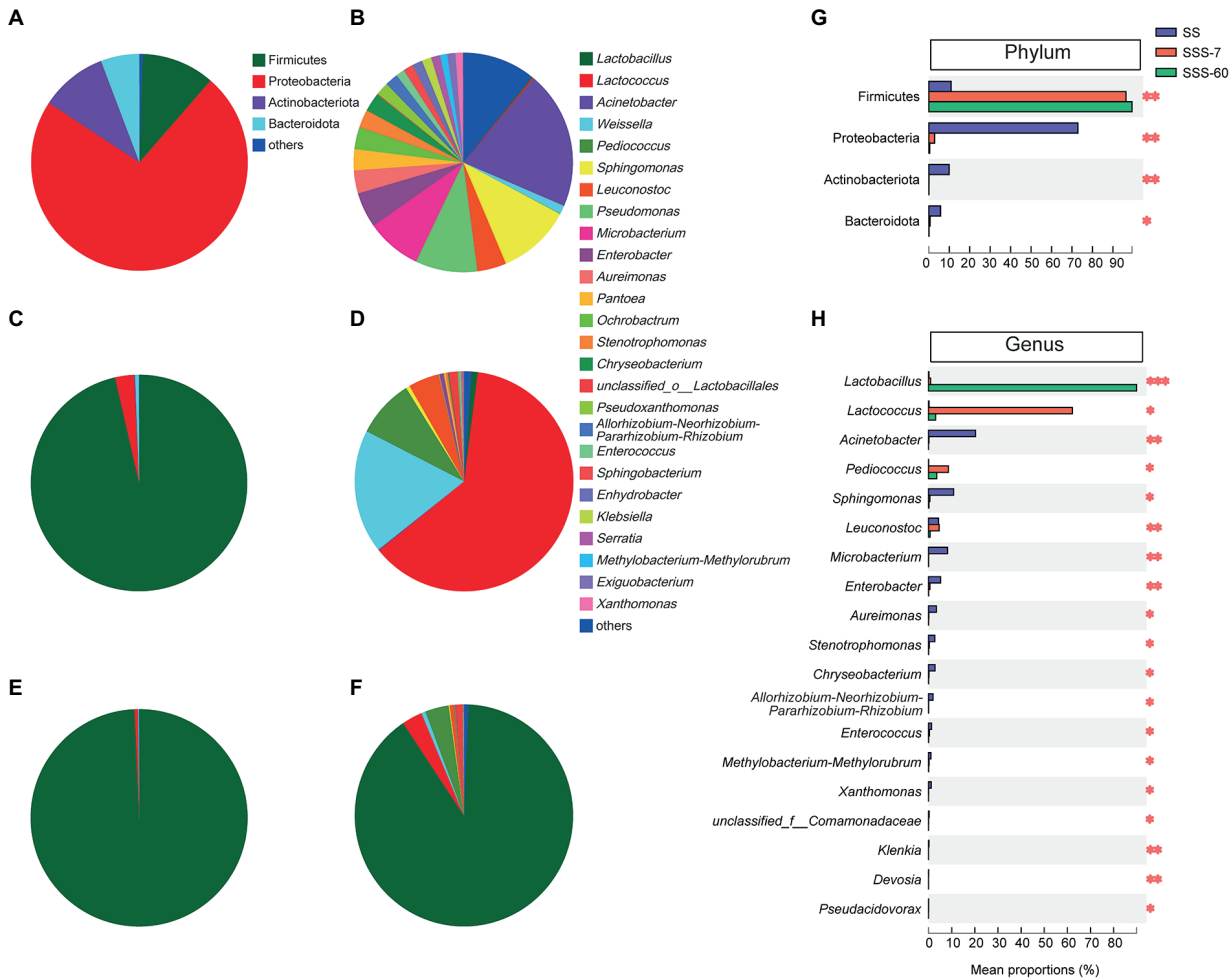
The pie plot of bacterial community at the phylum level in SS **(A)**, SSS-7 **(C)**, and SSS-60 **(E)**. The pie plot of bacterial community at the genus level in SS **(B)**, SSS-7 **(D),** and SSS-60 **(F)**. Multispecies difference test histogram of bacterial community at the phylum **(G)** and genus **(H)** level in SS and SSS. SS, high-moisture sweet sorghum; SSS, SS silage.

Figure 3A shows that Proteobacteria (72.9%), Firmicutes (10.9%), and Actinobacteriota (9.96%) were the phyla with high relative abundance (RA) in SS. After 7-day ensiling, the significant (*p* < 0.01) increase in RA of Firmicutes was accompanied by the significant (*p* < 0.05) decrease in RA of Proteobacteria, Actinobacteriota, and Bacteroidota (Figures 3C,G). After 60-day ensiling, Firmicutes were the predominant phylum (> 99%, Figures 3E,G).

There were 21 genera with a RA greater than 1% in SS (Figure 3B). The most abundant genus in SS was *Acinetobacter* (20.3%), followed by *Sphingomonas* (10.9%), *Pseudomonas* (9.15%), and *Microbacterium* (8.17%). As ensiling proceed, the RA of *Acinetobacter*, *Sphingomonas*, *Microbacterium*, and *Enterobacter*, etc. significantly (*p* < 0.05) decreased, while the RA of *Lactobacillus*, *Lactococcus,* and *Pediococcus* significantly (*p* < 0.05) increased (Figure 3H). In SSS-7, *Lactococcus* (62.2%), *Weissella* (18.2%), and *Pediococcus* (8.60%) were the advantage genera (Figure 3D). And in SSS-60, *Lactobacillus* (89.9%) predominated its bacterial community (Figure 3F).

### Correlation analysis of fermentation parameters and bacterial community

**Figure 4:**
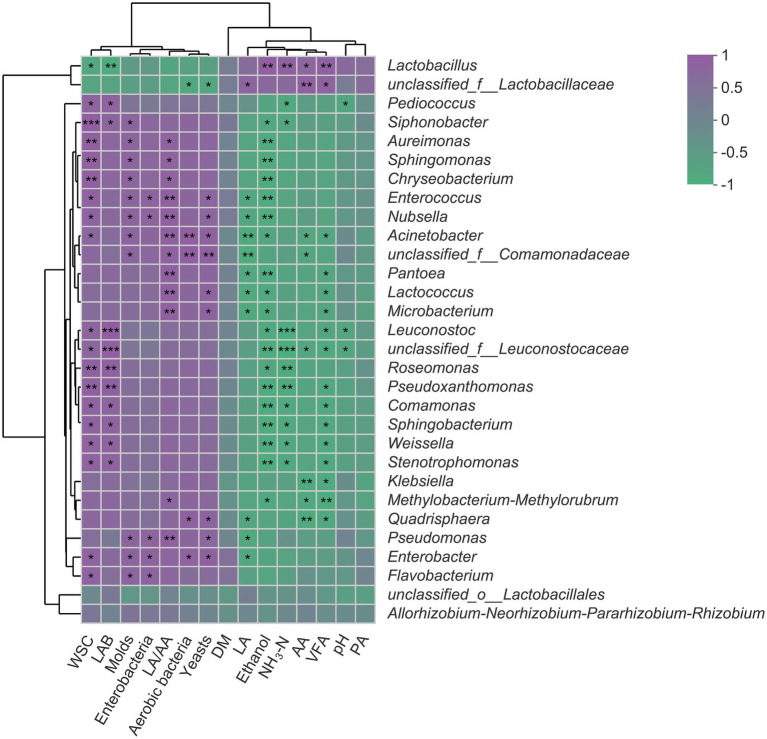
Spearman’s correlation heatmap of fermentation parameters and top 30 genera in SSS. Purple squares refer to positive correlation (0 < *r* < 1), whereas green squares refer to negative correlation (−1 < *R* < 0). ^*^p < 0.05; ^**^0.001 < *p* ≤ 0.01; ^***^p ≤ 0.001. WSC, water-soluble carbohydrates; LAB, lactic acid bacteria; LA/AA, the ratio of lactic to acetic acid; DM, dry matter; LA, lactic acid; NH_3_-N, ammonia nitrogen; AA, acetic acid; VFA, volatile fatty acid; PA, propionic acid.

The Spearman’s correlation analysis of fermentation parameters and bacterial community is visualized through a heatmap (Figure 4). The RA of *Lactobacillus* was positively correlated with AA and VFA but negatively correlated with WSC content (*p* < 0.05). Also, there were negative correlations between RA of *Lactobacillus* and the number of aerobic bacteria, yeasts, molds, and enterobacteria. The RA of *Pediococcus* and *Leuconostoc* was negatively correlated with pH value and NH_3_-N concentrations while positively correlated with WSC content (*p* < 0.05). The concentrations of LA, AA, PA, and VFA were negatively correlated with the RA of all detected genera (top 30) except *Lactobacillus* and unclassified_f__*Lactobacillaceae*.

### Kyoto encyclopedia of genes and genomes (KEGG) functional prediction of bacterial community

**Figure 5:**
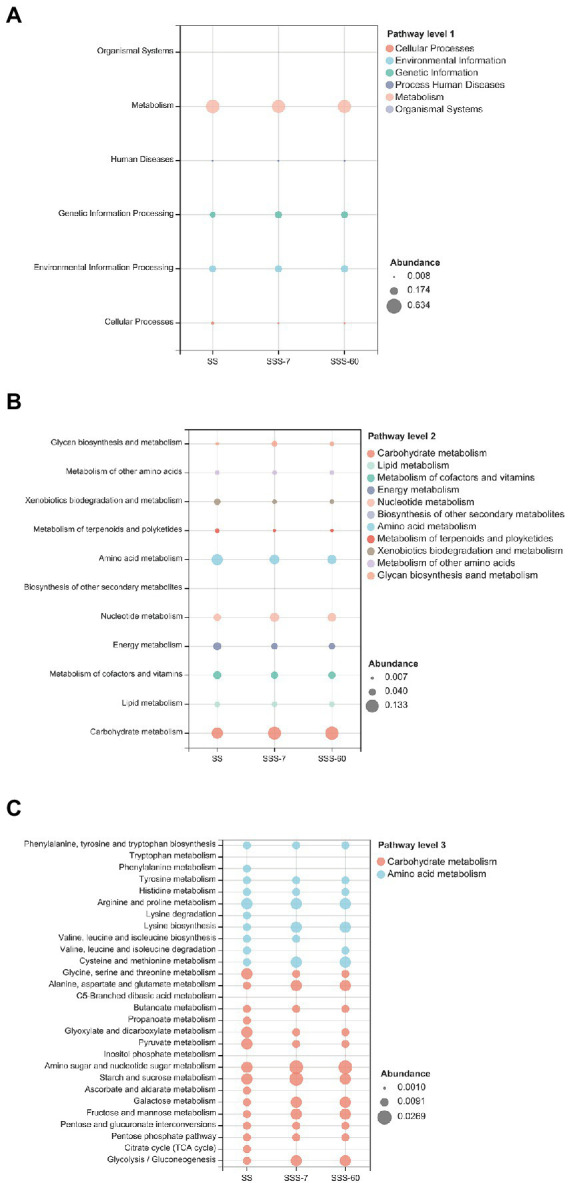
16S rRNA gene-predicted Kyoto Encyclopedia of Genes and Genomes (KEGG) function profiles at pathway level 1 **(A)**, pathway level 2 **(B)** and pathway level 3 **(C)**. SS, high-moisture sweet sorghum; SSS, SS silage.

As shown in Figures 5A,B, the KEGG function profiles under pathway level 1 in SS and SSS were primarily associated with metabolism, and the function profiles under pathway level 2 were mainly related to carbohydrate metabolism and amino acid metabolism, then nucleotide metabolism, metabolism of cofactors and vitamins, and energy metabolism. The abundance of those common functional categories was different between SS and SSS (SSS-7 and SSS-60). Specifically, SSS-60 had the highest abundance of carbohydrate metabolism, followed by SSS-7 and SS. Whereas, the abundance of amino acid metabolism exhibited the opposite tendency, with the highest value in SS and lowest value in SSS-60.

Under pathway level 3 (Figure 5C), the carbohydrate metabolism including glycolysis/gluconeogenesis, pentose phosphate pathway, pentose and glucuronate interconversions, fructose, mannose, galactose, starch, sucrose, amino sugar, and nucleotide sugar metabolism was upregulated in SSS compared with SS, while citrate cycle (TCA cycle), ascorbate, aldarate, inositol phosphate, pyruvate, glyoxylate, dicarboxylate, propanoate, butanoate, and C5-Branched dibasic acid metabolism were downregulated in SSS in comparison with SS. The amino acid metabolism including glycine, serine, threonine, arginine, proline, histidine, phenylalanine, and tryptophan as well as valine, leucine, isoleucine, and lysine degradation was downregulated in SSS in comparison with SS, while alanine, aspartate, glutamate, cysteine, methionine, and tyrosine metabolism and lysine biosynthesis were upregulated in SSS compared with SS.

## Discussion

### Analysis of raw material

For ensiling, the WSC content and epiphytic LAB number are the key factors determining the quality of silage. In the present study, the WSC content (> 5% DM) and LAB number (> 5.0 log_10_ CFU/g FM) of SS far satisfied the requirement for well-preserved silage (McDonald et al., 1991; Cai et al., 1999), but the low DM content and high yeasts and enterobacteria number (> 6.0 log_10_ CFU/g FM) might pose challenges to the production of high-quality silage.

### Effects of ensiling time on fermentation quality of SSS

According to Kung and Shaver (2001), the pH value of high-quality silage should be lower than 4.20 within 21 days of ensiling. The rate of pH decline during ensiling was then used to determine whether the silage was preserved effectively. The faster the pH declines, the less the risk of adverse microbial activities and nutrient losses in silage. In this study, the fast acidification of SSS was due to the high WSC content, sufficient LAB number, and low BC of SS. Similar to most studies, AA and VFA concentrations increased with the extension of ensiling, and this could be associated with the activities of AA-producing microbes and the proliferation of heterofermentative LAB. The LA/AA of all SSS was greater than 2.0, suggesting that the fermentation pattern of SSS in this study was the homolactic type (Kim et al., 2017).

The propionate, butyrate, and ammonia nitrogen concentrations are the main negative factors affecting silage fermentation (Kung et al., 2018). In the present study, the final PA concentration (0.19% DM) was within the acceptable level (< 1% DM; Agarussi et al., 2019). Meanwhile, no BA (n-BA and IBA) was detected throughout the ensiling, indicating that there was no clostridial fermentation in SSS. Further, the final NH_3_-N concentration of SSS (7.59% TN) was no more than the maximum limit (10% TN) for well-preserved silage (McDonald et al., 1991), suggesting that no serious proteolysis occurred in SSS. The acceptable PA, BA, and NH_3_-N concentrations of SSS could be attributed to the rapid acidification in the initial phase of ensiling, which effectively inhibited the activities of the related microbes and enzymes.

The fast proliferation of LAB in the initial phase of ensiling was because the high WSC content of SS ensured the full growth of epiphytic LAB. Unsurprisingly, the number of aerobic bacteria and molds that cannot tolerate anaerobic conditions continuously decreased to a negligible or undetectable level as ensiling proceeded and O_2_ depleted. And the negligible or undetected number of yeast and enterobacteria in SSS was associated with the low pH value (Zhao et al., 2022). Considering that yeast is more resistant to acid, the decrease of yeasts in SSS was also related to the rapid proliferation of LAB as the former are less competitive under anaerobic and acidic conditions (McDonald et al., 1991; Zhao et al., 2021).

### Effects of ensiling time on the bacterial community of SSS

The average coverage index in all sequenced samples was above 99%, suggesting that the sequencing depth was reliable for bacterial community analysis (Dong et al., 2020). Mendez-Garcia et al. (2015) stated that low pH conditions are mainly responsible for the reduced microbial diversity in acidic habitats. Similarly, in this study, the low pH value after ensiling decreased the bacterial α-diversities of SS, and this could be associated with the disappearance of acid-intolerant aerobic bacteria (Dong et al., 2020).

The PCoA plot was constructed to visualize bacterial community composition differences as distances between treatments. The clear separation of symbols between SS and SSS confirmed great differences in the bacterial community composition of SS before and after ensiling, which was attributed to the disappearance of and acid-intolerant aerobic bacteria during ensiling as abovementioned. Among them, the symbols of SSS-7 and SSS-60 were also well separated, which suggested the obvious bacterial community differences from the initial to the late phase of ensiling.

The obvious shift of the bacterial community from Proteobacteria to Firmicutes after ensiling could be attributed to the effective inhibition of aerobic bacteria (*Acinetobacter*, *Sphingomonas*, *Aureimonas*, etc.) and the obvious proliferation of LAB (*Lactobacillus*, *Lactococcus,* and *Pediococcus*). The ensiling process promoted the growth of Firmicutes because they are typical microbes in anaerobic and acid environments (Zhao et al., 2017). For quality silage, LAB including *Lactococcus*, *Pediococcus*, and *Lactobacillus* should be dominant in the bacterial community during ensiling. It is generally accepted that the early colonization of cocci-shaped LAB (*Lactococcus*, *Pediococcus*, and *Enterococcus*) is favorable for the establishment of an initial acidic environment and the growth of rod-shaped LAB (*Lactobacillus*), which is the prerequisite to ensuring the quality of silage (Muck, 2013; Graf et al., 2016). Similarly, with the process of ensiling, the bacterial community of SSS was firstly dominated by *Lactococcus*, *Weissella*, *Pediococcus*, and *Leuconostoc* and finally dominated by *Lactobacillus*.

### Correlation of fermentation parameters and bacterial community of SSS

The positive correlation between *Lactobacillus* and AA and the negative correlation between *Lactobacillus* and WSC suggested that this genus was primarily responsible for the acetate generation and sugar consumption in SSS. It would be desirable to see that the WSC is mainly used by LAB rather than harmful bacteria, as this can quickly establish an acidic environment and greatly avoid the unnecessary loss of substrate, which, in turn, explained the negative relationships between *Lactobacillus* and aerobic bacteria, yeasts, molds, and enterobacteria. *Pediococcus* and *Leuconostoc* as early colonizers are crucial for the initial pH decline (Cai et al., 1998; Yang et al., 2019), and this accounted for their negative correlations with pH and NH_3_-N because the rapid acidification effectively inhibited the proteolytic bacteria and plant enzyme, thereby reducing the formation of NH_3_-N. The negative correlations between fermentation products (LA, AA, PA, and VFA) and most detected genera further confirmed that short-chain fatty acids have a broad spectrum of bacteriostatic properties (Yuan et al., 2018).

### Effects of ensiling time on the potential function of the bacterial community in SSS

The NGS is not only a powerful tool to assess microbial community structure and diversity, but also provides insights into the metabolic potential of the community, which is crucial for the analysis of microbial community (Asshauer et al., 2015). In recent years, the PICRUSt and Tax4fun tools have been successively proposed to predict the KEGG function profiles of microbial communities using 16S rRNA gene sequences. Here, Tax4fun was applied to predict the KEGG functional profiles of the bacterial community in SS and SSS due to its superior performance to PICRUSt. Based on the abundance, the functional profiles under pathway level 2 of the microbiota involved in silage fermentation are dominated by the metabolism of carbohydrate, amino acid, nucleotide, cofactors, vitamins, and energy, which is in line with the finding of Bai et al. (2021) The principle of ensilage is that the available carbohydrates in forage are fermented to short-chain fatty acids (SCFAs, mainly lactate) by LAB under anaerobic conditions, which may explain the higher abundance of carbohydrate metabolism after ensiling. As essential substances in plants, amino acids play an important role in promoting primary metabolism and protein synthesis, that is why the abundance of amino acid metabolism in the SS is high. While the suppressed amino acid metabolism in SSS could be attributed to the pH declines. Flythe and Russell (2004) reported that the acidic environment established by ensiling can inhibit the amino acid metabolism of forage. The metabolism of cofactors and vitamins was suppressed after ensiling, which was consistent with the results of Wang et al. (2022) Although vitamins in forage (e.g., α-tocopherol and β-carotene) are beneficial to the immune system of ruminants, they can be degraded during ensiling (Liu et al., 2019). According to Bai et al. (2021), the upregulated metabolism of cofactors and vitamins assisted by specific LAB is beneficial to the production of vitamins during ensiling. Hence, it is necessary to ensile SS with some functional LAB to reduce the vitamin loss. As reported by Pessione et al. (2010), the energy metabolism of LAB (mainly malate decarboxylation, amino acid decarboxylation, and arginine deamination) is crucial for promoting LA production in silage. Xu et al. (2021) stated that energy metabolism should be promoted in quality silage. Strangely, the ensiling process did not promote the energy metabolism of SSS, even if they were well-fermented. Furthermore, nucleotides can be used to synthesize DNA and provide major energy for cellular processes (Kilstrup et al., 2005). Intriguingly, the shifts of nucleotide metabolism in this study were opposite to that of energy metabolism. Thus, other omics methods, such as proteomics, metagenomics, and metabolomics, are required to further clarify the functional annotation and metabolic pathway of the microbial community before and after ensiling.

To further clarify the functional shifts of the bacterial community in SS and SSS, the carbohydrate metabolism and amino acid metabolism were specifically analyzed under pathway level 3. The metabolism of most carbohydrate components (amino sugar, nucleotide sugar, sucrose, etc.) was promoted with the process of ensiling, which confirmed that LAB can utilize a variety of carbohydrate sources for their proliferation. Among them, the high abundance of fructose, mannose, galactose, sucrose, amino sugar, and nucleotide sugar metabolism in SSS are primarily related to *Lactobacillus*, *Lactococcus*, *Weissella,* and *Pediococcus*. Differently, the TCA cycle, ascorbate, aldarate, inositol phosphate, pyruvate, glyoxylate, dicarboxylate, propanoate, butanoate, and C5-branched dibasic acid metabolism were suppressed by ensiling. The suppression of the TCA cycle after ensiling could ascribe to the depletion of O_2_ in silos, as this cycle must be performed under aerobic conditions (Banfalvi, 1991). It is known that the Embden–Meyerhof–Parnas (EMP) pathway, the most common type of glycolysis, assists homofermentative LAB to ferment glucose into LA. Hence, we speculated that the higher abundance of glycolysis after ensiling may be related to the abundant homofermentative LAB. Meanwhile, the higher RA of *Leuconostoc* and *Weissella* after ensiling might explain the higher abundance of pentose phosphate (PPP) pathway, because heterofermentative LAB possesses the PPP (Abdel-Rahman et al., 2011). Amino acid metabolism, especially amino acid degradation, is of significance to explain the formation of NH_3_-N during ensiling. And the acceptable NH_3_-N content (< 100 g/kg TN) in this study might be due to the suppression of valine, leucine, isoleucine, and lysine degradation.

## Conclusion

The SS contains high WSC content and sufficient LAB number, which can be used as a promising alternative to silage maize for silage production. The ensiling process had remarkable effects on fermentation parameters, bacterial community, and functional profiles of sweet sorghum silage. The study could be a reference for providing comprehensive insights into the fermentative mechanism of silage through NGS and KEGG functional prediction analyses.

## Data availability statement

The data presented in the study are deposited in the Sequence Read Archive (SRA), accession number PRJNA843380. We have released the above data.

## Author contributions

JZ and TS conceived and designed the research, contributed to manuscript revision, read, and approved the submitted version. JZ and XJ conducted experiments. JL and SW contributed analytical tools. JZ and ZD analyzed the data. JZ wrote the first draft of the manuscript. All authors contributed to the article and approved the submitted version.

## Funding

This study was financially supported by the Joint Fund for Regional Innovation and Development of the National Natural Science Foundation of China (U20A2003).

## Conflict of interest

The authors declare that the research was conducted in the absence of any commercial or financial relationships that could be construed as a potential conflict of interest.

## Publisher’s note

All claims expressed in this article are solely those of the authors and do not necessarily represent those of their affiliated organizations, or those of the publisher, the editors and the reviewers. Any product that may be evaluated in this article, or claim that may be made by its manufacturer, is not guaranteed or endorsed by the publisher.
